# Use and Uptake of eHealth in General Practice: A Cross-Sectional Survey and Focus Group Study Among Health Care Users and General Practitioners

**DOI:** 10.2196/medinform.4515

**Published:** 2016-04-06

**Authors:** Jose M Peeters, Johan W Krijgsman, Anne E Brabers, Judith D De Jong, Roland D Friele

**Affiliations:** ^1^ NIVEL Utrecht Netherlands; ^2^ Nictiz Den Haag Netherlands; ^3^ Netherlands Institute for Health Services Research (NIVEL) Utrecht Netherlands

**Keywords:** eHealth, technology, GPs, general practice, implementation, survey, health care users

## Abstract

**Background:**

Policy makers promote the use of eHealth to widen access to health care services and to improve the quality and safety of care. Nevertheless, the enthusiasm among policy makers for eHealth does not match its uptake and use. eHealth is defined in this study as “health services delivered or enhanced through the Internet and related information and communication technologies.”

**Objective:**

The objective of this study was to investigate (1) the current use of eHealth in the Netherlands by general practitioners (GPs) and health care users, (2) the future plans of GPs to provide eHealth and the willingness of health care users to use eHealth services, and (3) the perceived positive effects and barriers from the perspective of GPs and health care users.

**Methods:**

A cross-sectional survey of a sample of Dutch GPs and members of the Dutch Health Care Consumer Panel was conducted in April 2014. A pre-structured questionnaire was completed by 171 GPs (12% response) and by 754 health care users (50% response). In addition, two focus groups were conducted in June 2014: one group with GPs (8 participants) and one with health care users (10 participants).

**Results:**

Three-quarters of Dutch GPs that responded to the questionnaire (67.3%, 115/171) offered patients the possibility of requesting a prescription via the Internet, and half of them offered patients the possibility of asking a question via the Internet (49.1%, 84/171). In general, they did intend to provide future eHealth services. Nonetheless, many of the GPs perceived barriers, especially concerning its innovation (eg, insufficient reliable, secure systems) and the sociopolitical context (eg, lack of financial compensation for the time spent on implementation). By contrast, health care users were generally not aware of existing eHealth services offered by their GPs. Nevertheless, half of them were willing to use eHealth services when offered by their GP. In general, health care users have positive attitudes regarding eHealth. One in five (20.6%, 148/718) health care users perceived barriers to the use of eHealth. These included concerns about the safety of health information obtained via the Internet (66.7%, 96/144) and privacy aspects (55.6%, 80/144).

**Conclusions:**

GPs and health care users have generally positive attitudes towards eHealth, which is a prerequisite for the uptake of eHealth. But, general practitioners in particular perceive barriers to using eHealth and consider the implementation of eHealth to be complex. This study shows that there is room for improving awareness of eHealth services in primary care. It will take some time before these issues are resolved and eHealth can be fully adopted.

## Introduction

Support among national policy makers and health officials for eHealth in many Western countries is considerable, and efforts are focused on national strategies to expand its use [1]. Active promotion of eHealth arises from the belief that eHealth widens access to health care services and has considerable potential to increase service efficiency [1-3]. Furthermore, the use of eHealth has the potential to support patients’ self- management, especially in those with chronic diseases such as asthma [4]. eHealth also has a potentially considerable impact on the use of health systems and patient-doctor roles [5].

There are many different definitions of eHealth in the literature [6,7]. A commonly used definition of eHealth is “health services and information, delivered through the Internet and related information and communication technologies, to improve or to enable health and health care” [8]. We use this definition in our study, which focuses on the use of patient online services in primary care for example, making an appointment with the general practitioner (GP) via the Internet and asking the GP a question via the Internet.

The global use of Internet has expanded dramatically in the last 10 years [9]. More than 90% of GPs offer Internet services that can be used by patients to communicate with their practice [10]. In European primary care, positive evolution in the use of eHealth is clearly observable. For example, the use of electronic networks for the transmission of medical patient data is well established and widespread. But the enthusiasm for eHealth among national policy makers is generally not matched by uptake and use in primary care among GPs and health care users [10,11].

From previous research, we know that the introduction of eHealth services is often seen as disruptive in relation to existing practice, rather than being supportive [2,3,12,13]. New systems and technologies also arrive with a set of assumptions of user needs, and they may not match user views and expectations [14,15]. We also know that beliefs and attitudes play an important role in the adoption of technology [16-18].

There is considerable literature available about the adoption of innovations in general, and in many disciplines such as public health [19]. However, to our knowledge, less is known about more specific areas, such as the process of adopting eHealth services in general practice, which is the focus of our study. The implementation of Internet communication services in primary care by GPs is expected to have positive effects because these services can increase the efficiency of care, patient satisfaction, and quality of care. Studying eHealth use in the area of primary care is important, as this may generate invaluable knowledge, for instance about access to primary care. Information about access is also important because of the clear gate-keeping role of GPs for (more expensive) medical specialists in the health care system. For example, the use of online communication (e-consults) by GPs in primary care practice can reduce the number of office visits and can enlarge primary care access [10]. This is the case in the Netherlands, where the GP is the entry point to the system.

The aim of this descriptive study is to gain insight into the current use of eHealth services by GPs and health care users and to identify the needs and perceived barriers of GPs and health care users using eHealth. This paper addresses the following questions for GPs: (1) What eHealth services do GPs currently provide? (2) What eHealth services do GPs intend to provide in future? (3) What are the needs and barriers that GPs face in providing current/future eHealth services? We also address similar questions for health care users: (1) What services do health care users currently use? (2) What services are health care users willing to use in future? (3) What are the needs and barriers that health care users face in using current/future eHealth services? The findings from the perspective of both GPs and health care users will enable us to compare both perspectives. This examination can contribute to the implementation of eHealth and the uptake of eHealth use in general practice.

## Methods

### Survey

This study is part of an annual, national survey about eHealth in the Netherlands, “The eHealth-monitor”, financed by the Dutch Ministry of Health, Welfare and Sport [20]. In 2014, the monitor study was performed for the second time and these data are used in this paper [21].

### Recruitment

#### General Practitioners

In April 2014, we sent an online questionnaire to a sample of 1402 GPs. These GPs were drawn from the members of the Royal Dutch Medical Association, which is representative of Dutch GPs in age and gender. At the time of the study, about 12,400 GPs were members of the Royal Dutch Medical Association. We sent an email reminder at 2 weeks and 4 weeks. In total, 171 GPs completed the questionnaire.

#### Health Care Users

In April 2014, a sample of 1500 panel members of the Dutch Health Care Consumer Panel run by NIVEL (Netherlands Institute for Health Research) was drawn. This sample was representative of the Dutch population aged 18 years and older regarding gender and age. This consumer panel is an access panel that consists of a large number of individuals who have agreed to answer questions on a regular basis. At the time of the study, the panel comprised approximately 6750 members [22]. The collected data are protected by registration with the Dutch Data Protection Authority (No. 1262949). In addition, the panel has privacy regulations.

We sent questionnaires by post or email, according to the respondents’ previously stated preference. After 2 weeks, a postal reminder was sent. Those respondents who preferred to fill in an online questionnaire received a reminder after 1 week and again after 2 weeks by email. A total of 754 health care users filled out the questionnaire.

### Focus Groups

In June 2014, two focus groups were set up: one with GPs and one with health care users. The GPs were recruited from the respondents who gave permission in the questionnaire to receive an invitation for a focus group. The health care users were recruited from the respondents who gave permission in the questionnaire to receive an invitation for a focus group. Eight practicing GPs attended the focus group (2 women, 6 men). The focus group of health care users consisted of 10 individuals (5 women and 5 men). We did not ask the participants for their age.

The goal of the focus groups was to obtain feedback on the results of the survey and was meant to complement the quantitative part of the study. The goal of the focus groups was to gain more insight into the motives and underlying reasons for the participants to use (or not use) eHealth services and examine which positive effects and which barriers they perceive regarding the use of eHealth.

### Questionnaire

#### General Practitioners

We asked GPs how often they use the Internet in their daily work and which device they use to access the Internet. We also asked the GPs (1) which eHealth services they currently offer in their general practice (eg, making an appointment with the GP via the Internet, (2) their plans to offer eHealth services in future, and (3) their perceived barriers to offering eHealth services.

#### Health Care Users

The questionnaire for health care users addressed the same eHealth-related topics as those in the questionnaire for GPs. Questions were asked about the use of Internet at home, for example, for gathering information about health and health care: (1) familiarity with eHealth services, offered by their GP, (2) usage and willingness to use eHealth services, and (3) perceived barriers to using eHealth. Only the respondents who had contact with their GP during the past year were asked to answer the questions about familiarity with eHealth and willingness to use eHealth.

### Analysis

#### Questionnaire

To describe the use and the perceived barriers of eHealth services by health care users and by GPs, we used descriptive statistics. The analyses for the GPs were performed with the statistical program SPSS, version 19.0. The analyses for health care users were performed with the statistical program Stata, version 13.0.

For questions asked to all health care users, we weighted the descriptive analyses for age and gender in such a way that it resembled the distribution of age and gender within the Dutch population from 18 years, based on data from Statistics Netherlands. We applied a weighting factor ranging from 0.6 to 1.5.

The GP sample is representative of the Dutch population of GPs regarding gender, but the response is not representative for age: GPs younger than 35 years and GPs aged 50 years and older responded more often. Nevertheless, we did not use a weight factor to correct for this because applying the weight factor did not affect the results.

#### Focus Groups

In the two focus groups, the main results of the survey were discussed with the participants (GPs and health care users). The focus group feedback was recorded, transcribed, and coded in relevant topics.

## Results

### General Practitioners

The questionnaire was completed by 171 GPs, which is a 12% response rate (52.0%, 89/171 male; mean age 46 years, range 31-68 years). All the GPs in this study accessed the Internet in their daily work, using a computer or laptop (100.0%, 171/171), smart phone (80.7%, 138/171), or tablet (39.2%, 67/171). GPs used the Internet mostly to gather medical information (90%) or to show information to patients (78.9%, 135/171).

In this section we discuss the three research questions regarding GPs.

#### What eHealth Services Do GPs Currently Provide?

The possibility of requesting a prescription via the Internet was the most common eHealth service offered by GPs (see Table 1; 67.3%, 115/171). In second place, half of the GPs (49.1%, 84/171) stated that they offer patients the opportunity to ask them a question via the Internet. Other eHealth services, such as making an appointment via the Internet, receiving a reminder for an appointment, and screen-to-screen contact between GP and patient, were scarce (0.6%, 1/171 to 18.1%, 31/171).

#### What eHealth Services Do GPs Intend to Provide in Future?

GPs who do not have plans for offering eHealth services, often reported that they would like to offer these services. For example, four out of ten GPs (41.5%, 71/171) would like to offer patients the possibility of receiving a reminder for an appointment via the Internet or by text message (see Table 1). Looking at the plans and the willingness of the GPs to offer more eHealth services in the near future, we found that almost a quarter of the GPs plan to offer patients the opportunity to make an appointment via the Internet within 1 year (22.8%, 39/171).

#### What Are the Needs and Barriers Facing GPs in Providing Current or Future eHealth Services?

Most of the GPs (79.5%, 136/171) who completed the questionnaire experienced barriers regarding eHealth (see Table 2). About half of the GPs mentioned that communication with patients via the Internet is not explicit enough (48.5%, 66/136). They also noted that implementation of eHealth is time-consuming and that there is no funding or financial compensation for the effort and time they spend on it (48.5%, 66/136). GPs also perceived that contact by telephone or face-to-face contact is more efficient than contact via the Internet (42.6%, 58/136) and that they do not have the time for training or upskilling regarding eHealth (40%, 54/136).

The general experience of GPs in the focus groups was that the implementation of eHealth is inevitable. One GP stated that “eHealth is becoming more and more important, so I had better prepare for it.” All the participating GPs in the focus groups were familiar with eHealth, but they were also reluctant to use eHealth. “There is no triage when patients make an appointment via the Internet and there is no patient information available” (GP1)*.* GPs who attended the focus group also “fear loss of control of their agenda” (GP2) and “fear huge increase of patient appointments” (GP3). Providing patient online communication is also perceived as “time-consuming and expensive” (GP4), and “the reimbursement for an e-consult is not sufficient to compensate the investments” (GP5)*.*

**Table 1:** GPs who offer and are willing to offer eHealth-services in their general practice (N=171).

According to GPs	n (%)				
	This is offered	There are plans to offer within 1 year	There are no plans, but I would like to offer	There are no plans, and I do not know if I would like to offer	There are no plans, and I would not like to offer
To make an appointment with my GP via the Internet	31 (18.1)	39 (22.8)	44 (25.7)	40 (23.4)	17 (9.9)
To receive a reminder for an appointment with my GP via the Internet or text message	13 (7.6)	12 (7.0)	71 (41.5)	50 (29.2)	25 (14.6)
To ask my GP for a requesting prescription via the Internet	115 (67.3)	16 (9.4)	28 (16.4)	7 (4.1)	5 (2.9)
To ask my GP a question via the Internet	84 (49.1)	13 (7.6)	17 (9.9)	39 (22.8)	18 (10.5)
To talk with my GP screen to screen via the Internet, for example via a tablet	1 (0.6)	4 (2.3)	33 (19.3)	78 (45.6)	55 (32.1)

**Table 2:** Barriers to using eHealth, perceived by GPs (N=171).

Barriers		n (%)
**Perceived barriers (N=171)**		
	Yes	136 (79.5)
	I do not know	27 (15.8)
	No	8 (4.7)
**Type of barriers (N=136)**		
	The communication is not explicit enough, when contacting via the Internet	66 (48.5)
	Lack of financial fees for the time spent to implement eHealth	66 (48.5)
	Less efficient than contact by telephone or face-to-face contact	58 (42.6)
	Lack of time to delve into this	54 (39.7)
	Lack of sufficient safe systems	52 (38.2)
	Fear of criticism about privacy aspects	49 (36.0)
	Fear of increase in patients’ care demands	48 (35.3)
	Lack of clarity about laws and regulation regarding eHealth	46 (33.8)
	Doubts about the benefits for my general practice	46 (33.8)
	Lack of clarity about a good way to set up the system	40 (29.4)
	Fear that patients have higher expectations	38 (27.9)
	Lack of standards for the right set-up of systems	32 (23.5)
	Doubt about the benefits for patients	33 (24.2)
	Lack of knowledge and skills to apply eHealth in my general practice	32 (23.5)
	Lack of technical support	28 (20.6)
	Patients are unfamiliar with eHealth	24 (17.6)
	Resistance of employees in my general practice to expand the possibilities of eHealth	19 (13.9)
	Lack of opportunities for training	17 (12.5)
	No access to the right technique	14 (10.3)
	Patients’ resistance to expanding the possibilities for using eHealth	3 (2.2)

In the focus groups, GPs also reported that they “have a need for information about the do’s and the don’ts of eHealth, such as how to deal with privacy aspects or with triage when using electronic appointments” (GP6). Also, GPs in the focus groups mentioned that they “have an urgent need for information from a colleague GP” (GP7) so that they can learn from each other about how to deal with technical, financial, or organizational problems.

According to the focus groups, most of the GPs had plans to offer eHealth services in general practice because of the opportunities to widen access to their practice and to improve the service to patients. “The added value of providing online patient services is that the telephone of the general practice rings less often.” Another advantage for GPs was convenience: “I can answer patients’ online questions at a moment I prefer” (GP8)*.*

### Health Care Users

The questionnaire was completed by 754 members of the Dutch Health Care Consumer Panel, which is a response of 50% (51.1% male, 385/754; mean age 52 years, range 20-84 years). We also asked health care users questions about their Internet use at home because the availability and use of Internet is an important prerequisite for using eHealth. Almost all health care users (93.0%, 676/727) used the Internet at home, on various devices, such as a computer or a laptop (97.6%, 644/660), a smart phone (51.2%, 338/660), or a tablet (48.8%, 322/660). Many health care users (70.0%, 465/664) stated they find using the Internet easy, 20.0% (133/664) were neutral, and 9.9% (66/664) had the opinion that using the Internet is difficult. Health care users used the Internet especially for gathering information about health and health care (64.4%, 463/719), to look up information about nutrition and health (50.5%, 350/693), and to search for relevant information in deciding whether or not they should visit their GP (38.8%, 279/719).

In this section we answer the three research questions regarding health care users.

#### What eHealth Services Do Health Care Users Currently Use?

Table 3 shows that about half of health care users (48.6%, 282/580 to 60.0%, 352/587) who visited their GP last year at least once, did not know whether or not the above-mentioned eHealth services are offered by their GP. For example, 55.0% (323/587) did not know if it is possible to make an appointment via the Internet. Health care users were most familiar with the possibility of requesting a prescription from the GP via the Internet (30.5%, 177/580).

When we look at the frequency of eHealth use, 17.8% (102/573) of the health care users who visited their GP last year at least once used this eHealth service (see Table 4). Other eHealth services, such as making an appointment with the GP via the Internet and screen-to-screen contact between patient and GP, were hardly used in general practice, according to health care users (4.3%, 25/573 and 1.2%, 7/563, respectively; see Table 4).

#### What eHealth Services Are Health Care Users Willing to Use in Future?

About half of the health care users that did not use eHealth services reported that would like to use these services if offered by their GP (43.7%, 246/563 to 50.3%, 288/573; see Table 4). An exception is the possibility of talking with the GP via the Internet, for example a tablet. Only one out of five (19.0%, 107/563) would like to use this service if offered by their GP.

#### What Are the Needs and Barriers Facing Health Care Users in Using Current or Future eHealth Services?

One fifth of all the health care users (20.6%, 148/718) perceived barriers to using the Internet for their health and health care (Table 5). Health care users who perceived barriers mostly had “concerns about the validity of the information obtained via the Internet” (66.7%, 96/144) and “concerns about privacy aspects” (55.6%, 80/144). Barriers to eHealth use also had to do with beliefs. In this study, we found that half of the health care users thought that using the Internet was not suitable for personal contact (49.3%, 71/144). Health care users also needed more knowledge and skills in using eHealth (36.1%, 52/144), and they had doubts about the benefits of eHealth for themselves (35.4%, 51/144).

According to the focus groups, health care users also perceived benefits using eHealth. They were motivated to use eHealth for reasons of convenience, such as the possibility of contacting their GP at any time. Some members of the focus groups commented that “The use of Internet for health care is nice, but personal contact with the GP is also important” (PT1). “Internet is no substitute for personal care. Sometimes you want to speak your GP face-to-face” (PT2). Another member of the focus groups noted: “Change will occur slowly, because the privacy aspect and safety are also issues that should be addressed” (PT3).

**Table 3:** Familiarity of eHealth in general practice by health care users, who visited their GP at least once last year (N=580-587).

According to health care users	n (%)		
	This is possible	I do not know if it is possible	This is not possible
To make an appointment with my GP via the Internet	77 (13)	323 (55)	187 (32)
To receive a reminder for an appointment with my GP via the Internet or text message	31 (5)	352 (61)	197 (33)
To ask my GP for a requesting prescription via the Internet	177 (30)	282 (48)	127 (22)
To ask my GP a question via the Internet	84 (14)	340 (58)	159 (27)
To talk with my GP screen to screen via the Internet, for example via a tablet	8 (1)	348 (60)	225 (39)

**Table 4:** Use and willingness to use eHealth by health care users who visited their GP at least once last year (N=563-573).

According to health care users	n (%)			
	I used it, at least once last year	I did not use, but I would like to use	I did not use, and I do not know if I would like to use	I did not use, and I would not like to use
To make an appointment with my GP via the Internet	25 (4)	262 (46)	139 (24)	145 (25)
To receive a reminder for an appointment with my GP via the Internet or text message	13 (2)	261 (46)	125 (22)	166 (29)
To ask my GP for a prescription via the Internet	102 (18)	288 (50)	90 (16)	93 (16)
To ask my GP a question via the Internet	22 (4)	246 (44)	135 (24)	161 (29)
To talk with my GP screen-to-screen via the Internet, for example via a tablet	7 (1)	107 (19)	183 (32)	270 (48)

**Table 5:** Barriers to using eHealth, perceived by health care users (N=718).

Barriers		n (%)
**Perceived barriers by health care users (N=718)**		
	Yes	148 (20.6)
	I do not know	184 (25.6)
	No	386 (53.8)
**Type of barriers (N=144)**		
	Concerns about the validity of health information obtained via the Internet	96 (66.7)
	Concerns about privacy aspects	80 (55.6)
	Using the Internet is not suitable for personal contact	71 (49.3)
	Lack of knowledge and skills to adjust eHealth	52 (36.1)
	Doubt about the benefits of eHealth for myself	51 (35.4)
	Unfamiliarity with the possibilities of eHealth	45 (31.3)
	Lack of technical support	20 (13.9)
	Lack of time to delve into eHealth	18 (12.5)
	My care provider does not offer the opportunity	13 (9.0)
	I have no access to the Internet	7 (4.9)

## Discussion

### Principal Results

The results of the 2014 eHealth monitor show that three-quarters of the GPs that responded (67.3%, 115/171) offered patients the possibility of requesting a prescription via the Internet and half offered patients the possibility of ask them a question via the Internet (49.1%, 84/171). eHealth services for patients such as making an appointment via the Internet, receiving a reminder for an appointment, and screen-to-screen contact are much less likely to be offered by GPs. In general, the GPs in our study did have plans to offer eHealth services or at least they were willing to offer eHealth. Thus, the potential for further growth of eHealth services in general practice exists. However, we found that over three-quarters of respondents experience barriers to successful use eHealth. The main barriers they cited are communication problems, lack of financial compensation, and lack of time and technical skills to implement eHealth in daily practice. Accordingly, these barriers could hinder the further development of eHealth services.

The results of this survey also showed that eHealth services offered by GPs are not well known to health care users who had contact with their GP at least once last year. But nearly half of health care users are willing to use eHealth services, if offered by their GP, which means there is great potential for eHealth in the future.

It is worth pointing out the differences in perception of eHealth services between health care users and GPs. When we compare health care users and GPs, we may conclude that GPs often report that they offer eHealth services, while many health care users are not aware of these services being offered. That said, we have to keep in mind the low response rate of GPs. Accordingly, there appears to be a substantial gap between the availability of eHealth services in general practice and health care users’ familiarity with the possibility of using eHealth offered by their GP. To increase familiarity with eHealth services, websites such as National Health Services Choices in England is an example of altering health care seeking behavior, attitudes, and knowledge among health care users [23].

When we compare the findings of our study about barriers perceived by GPs with those perceived by health care users, it is remarkable that only one fifth of health care users perceive barriers to using eHealth versus the majority of GPs. A possible explanation for this gap in perceived barriers is that health care users scarcely use eHealth services, so it is plausible that they do not know whether or not they perceive barriers.

### Comparison With Previous Studies

Earlier studies showed that, in a European primary care setting, positive evolution is clearly observable in GPs’ use of the Internet, mainly with regard to online medical information searches, use of electronic health care records, and to a lesser extent, electronic transfer of patient data [24].

GPs are also increasingly seeking out eHealth services, such as digital prescribing and email consultations, to improve patients’ access to health care, patients’ quality of care, and service efficiency [2,25]. For example, a recent study of electronic prescribing suggests that after the implementation, the appropriate prescribing in polymedicated patients improved [26]. This is in line with our findings that GPs are optimistic about the potential of eHealth to increase access to primary care and improve quality of care. Our findings that GPs and health care users experience barriers are also in line with research about health care innovation in general [19] and with the results of other reviews and longitudinal studies regarding factors that promote or inhibit the implementation of eHealth [2,3,24,27].

### Strengths and Limitations

The main strength of this study is that we used a large number of health care users, with a subsample of health care users who visited their GP last year, and a large sample of GPs. The combination of a survey and focus groups is also a strength. In the focus groups, the results of the survey were discussed and we obtained important background information in the respondents’ motives for (not) using and offering eHealth as well as insight into facilitators and barriers to eHealth implementation. Thus, we have gathered valuable information about eHealth services, through the eyes of GPs and health care users as well.

A limitation of this study is the low response by GPs, which might have influenced the results and which means that the results cannot be generalized to the whole population of GPs in the Netherlands. The reason for the low response is that in 2014 we could not approach the panel members of the Royal Dutch Medical Association, due to the transition to another information system. Our solution was to approach a large, representative sample of members of this association. But because these members are not members of a panel (available for participation in research), we expected a lower response than in 2013 (it was 49%). The low response rate of 2014 was disappointing. In addition, we asked non-responders why they did not fill in the questionnaire. The main reasons were that GPs are very busy and that they often get requests by email to complete questionnaires. 

We want to stress that a bias in the sample may have occurred, namely that responses may have mainly come from those health care users and GPs who are very positive about using the Internet and eHealth, as well as respondents who are very negative about this topic. Nevertheless, we also conducted focus groups with GPs to reflect on the results. This was very informative, shedding more light on GPs’ attitudes about eHealth and their reasons for offering, or not offering, eHealth services. In the focus groups, we asked the participants to clarify their attitude to eHealth. Both focus groups represented participants with a positive attitude as well as participants who were negative about eHealth. Thus, we may conclude that both proponents and opponents are at represented in the focus groups in this study.

### Conclusions

This study showed that many GPs want to offer eHealth services in the near future because of the positive effects they expect when offering eHealth, for example, to expand access to their general practice. By contrast, health care users are not aware of the existing eHealth services their GPs offer. Nevertheless, most of the health care users are willing to use eHealth services, when offered by their GP, but they are not actively looking for eHealth services. In general, health care users and GPs have positive attitudes regarding eHealth. Therefore, the results imply that there are opportunities to further expand eHealth in general practice.

In our study, GPs perceived barriers to offering eHealth, such as communication problems, insufficient technical support, lack of financial compensation for the extra time spent on the implementation of eHealth, and their lack of knowledge and skills to implement eHealth properly. Health care users also had concerns about the safety of the health information via the Internet and about privacy aspects regarding the use of eHealth. Offering eHealth services in general practice is complex. Until now, widespread adoption of eHealth in general practice has been challenging because many problems have to be overcome. Thus, there are also many conditions that should be fulfilled to implement eHealth successfully and there is still a long way to go before eHealth is fully integrated in primary health care.

According to the results of this study, there is room for improving awareness of eHealth services in primary care. Increasing user awareness might result in more insight into the perceived benefits to health care users. To promote and further increase the use of eHealth services in general practice, best practices should be widespread. GPs could act as ambassadors to promote the knowledge of GPs and health care users about eHealth services and show how to use eHealth in general practice.

## Abbreviations

GP: general practitioner
